# Physiotherapy Professionals: A Questionnaire for the Evaluation of Telerehabilitation Services in Egypt

**DOI:** 10.5195/ijt.2024.6654

**Published:** 2025-01-15

**Authors:** Olfat Ibrahim Ali, Omnia Mustafa Salem, Salma Ashraf Abdelaziz Mohamed, Sabah Abdlnaser Hassan Ali, Eman Ahmed Embaby

**Affiliations:** 1 Physical Therapy Program, Batterjee Medical College, Jeddah 21442, Saudi Arabia; 2 Basic Science for Physical Therapy, Faculty of Physical Therapy, Cairo University, Giza, Egypt; 3 Faculty of Physical Therapy, Cairo University, Giza, Egypt

**Keywords:** Attitude, Awareness, COVID-19 pandemic, Perception, Physiotherapists, Telerehabilitation

## Abstract

**Purpose:**

This study aimed to evaluate the utilization of telerehabilitation services in Egypt and to investigate the concerns and barriers faced by Egyptian physiotherapists for implementation.

**Methods:**

This cross-sectional study recruited 306 Egyptian physiotherapists who were asked to complete an online survey including questions about the utilization of telerehabilitation services, awareness, perception, and attitude.

**Results:**

A total of 299 physiotherapists completed the online survey. Within the sample, 38.5% utilized telerehabilitation at work. Telerehabilitation was used frequently to deliver patient advice (17.6%), follow-up (16%), and exercise prescription (15.2%). Pain (16.2%) was the most common outcome assessed utilizing telerehabilitation. Additionally, 85.3% of physiotherapists agreed that the inclusion of telerehabilitation during the rehabilitation program is effective. The main barriers to implementing telerehabilitation in Egypt were lack of awareness (59.9%) and technical issues (58.2%).

**Conclusion:**

This study sheds light on the trends and challenges in utilizing telerehabilitation and may help in shaping the future of telerehabilitation in Egypt.

One of the major goals a physiotherapist focuses on is to gain the functional movement that is essential for a healthy lifestyle. Physiotherapy aims to provide individuals with health promotion, prevention of injuries, therapeutic interventions, and rehabilitation programs to restore activities of daily living (ADL) and sustain functional abilities throughout life. Patients can get access to physiotherapy sessions through hospitals and private clinics. However, their adherence can be influenced by several factors and uncontrolled conditions leading to halting patients’ progress and complicating their conditions. Such conditions also affect communication between the patient and the physiotherapist, which necessitates the call for an efficient solution.

Technological developments, such as telerehabilitation, contributed to overcoming such limitations as online platforms may support underserved or disadvantaged areas as well as low- to middle-income countries by granting access to care and healthcare providers anywhere in the world (Dicianno et al., 2015; Zampolini et al., 2008).

Telerehabilitation is defined as the use of telecommunications technologies to deliver medical services including assessing, diagnosing, treating, and following up with patients especially those in geographically remote areas (Seelman & Hartman, 2009). It can be performed via videoconferencing, email, and texting (Peretti et al., 2017). The use of telerehabilitation before the COVID-19 pandemic had been steadily increasing over the last decade, but overall growth has been slow. In response to the SARS-CoV-2 crisis, there was a sharp rise in its implementation (Saaei & Klappa, 2021).

Telerehabilitation has shown high rates of satisfaction with several physiotherapy specialties such as neurological, cardio-respiratory, and musculoskeletal disorders. However, plenty of barriers were encountered through the process of adopting such an approach as low technology literacy, poor infrastructure, internet connectivity issues, lack of awareness, inadequate staff skills, and patient privacy concerns (Peretti et al., 2017).

A review of previous studies provides valuable insights into the factors influencing physiotherapists' acceptance and adoption of telerehabilitation. Some of the studies were conducted in countries such as Finland, Saudi Arabia, Kuwait, Ghana, Pakistan, Nigeria, India, Spain, Switzerland, South Africa, Australia, United States of America, and United Kingdom (Albahrouh & Buabbas, 2021; Almutairi, 2023; Aloyuni et al., 2020; Awotidebe et al., 2023; Buckingham et al., 2022; Davies et al., 2023; Fernández-Lago et al., 2023; Kumar et al., 2021; Partanen et al., 2023; Paul et al., 2024; Ramanandi, 2022; Rausch et al., 2021; Rowe & Sauls, 2020; Shalabi et al., 2022; Zahid et al., 2017). Yet there is a knowledge gap in the literature concerning Egyptian physiotherapists’ perception and willingness to utilize telerehabilitation as part of their work routine. Therefore, this study aimed to evaluate telerehabilitation services in Egypt and investigate the concerns and barriers Egyptian physiotherapists may face while implementing them.

## Methods

### Study design

A countrywide cross sectional anonymous online survey was carried out among Egyptian physiotherapists between October and November 2023. The present study was approved by the Ethical Committee of Faculty of Physical Therapy, Cairo University (P.T.REC/012/004886). Before providing access to the survey questions, all participants gave informed consent through the online survey.

### Participants

A combination of convenient and snowball sampling was used; from the communication network of the research team members, potential participants were recognized. Sequentially these participants were asked to post on the survey to other potential participants. A sample of physiotherapists who were licensed and engaged in clinical practice in Egypt at least for one year were eligible to participate despite their degree of experience with telerehabilitation. We defined an *a priori* period of 4 weeks (October-November 2023) for the survey to be available online.

### Instrument

A self-administered online survey was created using Google Forms. All questions on the adapted but validated questionnaire used in this study were derived from the literature review (Albahrouh et al., 2021; Aloyuni et al., 2020; Awotidebe et al., 2023; Bezuidenhout et al., 2022; Buckingham et al., 2022; D'Souza & Rebello, 2021; Fernandes et al., 2022; Klappa, 2021; Rowe & Sauls, 2020; Saaei & Klappa, 2021; Tsekoura et al., 2022). The questionnaire included 33 closed-ended questions that were organized into four sections: sociodemographic data, utilization, awareness and perception, and attitude toward telerehabilitation. In section two, the participants were given two options (yes/no) to answer question 9 about the use of digital health tools/telerehabilitation at work. If they answered “yes,” they could proceed to the rest of the survey. In case they chose “no,” they were redirected to question 16 which asked them about the reasons of not using tele/online therapy. Some consisted of a 5- point Likert score ranging from 1 (‘strongly agree’) to 5 (‘strongly disagree’), other questions allowed two response options (‘yes’/‘no’).

### Procedures

Prior to dissemination, the content validity of the questionnaire, including clarity and relevance, was tested among six expert physiotherapists with research and clinical experience in orthopaedic, neurological, and geriatric rehabilitation, with minor modifications made to order, wording and structure. Content validity of questionnaire items was determined using the content validity index (CVI). Experts were asked to independently rate each item for ‘relevance’ to the underlying construct. They rated each item using a 4-point Likert scale, ranging from 1 = ‘item is not relevant’ to 4 = ‘item is highly relevant’ using the CVI, and in a similar manner for ‘clarity’ of the items. The CVI for each item (I-CVI) was calculated by the proportion of experts that rated the item as 3 or 4 (i.e., item is relevant/clear) (Yusoff, 2019). Additionally, scale content validity index (S-CVI) was calculated using both the average of the I-CVI scores for all items on the scale (S-CVI/Ave) and the proportion of items on the scale that achieved a relevance scale of 3 or 4 by all experts (S-CVI/UA) (Polit & Beck, 2006). According to calculations above, it was found that I-CVI, S-CVI/Ave and S-CVI/UA were > 0.8, and thus the questionnaire achieved a satisfactory level of content validity. The internal consistency of the total scale had a Cronbach's alpha of 0.71. Further, the questionnaire was revised and piloted with all authors for usability and functional technicality. The survey was distributed to respondents through WhatsApp and Telegram group chats. The estimated time of completion was between 10 – 15 minutes. It was completely voluntary to participate in the survey, and participants could withdraw at any time without consequences. A copy of the questionnaire is included in the Appendix.

### Statistical Analysis

Data were entered and validated using Microsoft Excel, while the statistical analysis was done using the Statistical Package for the Social Sciences (SPSS, Windows version 25). All continuous quantitative data were presented in mean and standard deviation (SD), while categorical data were presented in frequencies and percentages.

## Results

### Demographic Data of the Participants

This web-based survey was completed by 299 physiotherapists; seven physiotherapists refused to participate. Approximately two-thirds of respondents were female (67.2%), held a bachelor's degree in physiotherapy as their highest degree of education (41.5%), came from Great Cairo (60.2%), reported ˂ 5 years of clinical experience (37.8%) and worked >40 hours (28.4%). The ages of 54.5% of participants ranged from 23–30 years, followed by 35.8% with an age range of 31–39 years. Participants who were 50 years old or greater represented the lowest percentage (1%) of the total participants. Additionally, 42.5% of the participants worked at teaching hospitals and institutes, 37.1% worked at a general hospital and 35.5% worked at a private hospital. As shown in Table 1, most participants specialized in orthopedics (65.2%) or sports (34.8%).

**Table 1:** Demographic Data of the Sample

Variable	Categories	Frequency
Age (years)	23–30	163 (54.5%)
	31–39	107 (35.8%)
	40–49	26 (8.7%)
	50–59	2 (0.7%)
	=>60	1 (0.3%)
Sex	Male	98 (32.8%)
	Female	201 (67.2%)
Education	Bachelor's	124 (41.5%)
	Master's	103 (34.4%)
	Doctor of Philosophy (PhD)	61 (20.4%)
	Doctor of Physical Therapy (DPT)	6 (2%)
	Diploma	5 (1.7%)
Governate	Great Cairo (Cairo–Giza–Qalubia)	180 (60.2%)
	Delta Governorates	56 (18.7%)
	Canal City Governorates	8 (2.7%)
	Upper Egypt Governorates	19 (6.4%)
	Alexandria	21 (7%)
	Sinai and Red Sea	1 (0.3%)
	Other	14 (4.7%)
Experience	5 years	113 (37.8%)
	5–10 years	84 (28.1%)
	11–15 years	57 (19.1%)
	>15 years	45 (15.1%)
Working hours	<10	37 (12.4%)
	10–20	61 (20.4%)
	21–30	63 (21.1%)
	31–40	53 (17.7%)
	>40	85 (28.4%)
Work setting	Teaching hospitals/institute	127 (42.5%)
	General hospital	111 (37.1%)
	Private	106 (35.5%)
	Other	58 (19.4%)
Area of expertise	Cardiothoracic	27 (9%)
	Ergonomics	25 (8.4%)
	Geriatric rehabilitation	40 (13.4%)
	Integumentary and post–burn rehabilitation	21 (7%)
	Neurology	80 (26.8%)
	Nutrition	56 (18.7%)
	Oncology	5 (1.7%)
	Orthopaedic	195 (65.2%)
	Pediatrics	71 (23.7%)
	Sports	104 (34.8%)
	Women's Health	36 (12%)
	Not applicable	0 (0%)

### Utilization of Telerehabilitation Services

A total of 116 respondents (38.5%) used digital health tools at work compared to 185 respondents (61.5) who did not. For those who used digital health at work, 57 (49.2%) started utilization during the COVID pandemic; the first use of 31 (26.7%) was before pandemic and 28 (24.1%) began use of the digital tool after pandemic. Moreover, for participants using digital tools most (82.8%) used telepractice for less than 5 years, and fewer (14.7%) for 5–10 years. Among those who use digital tools at work, 23.3% used them every day, 36.2% used them several days per week and 40.5% used them several days per month. The greatest use of digital tools was for advice (17.6%) followed by follow up treatment (16%), exercise prescription (15.2%) and assessment (11.4%). Pain (16.2%), range of motion (14.8%), and posture (14.6%) were the more common outcomes assessed using digital tools (see Table 2).

**Table 2:** Utilization of Telerehabilitation Services

	Category	Frequency (%)
9. Do you use digital health tool at work?	Yes	116 (38.5%)
	No	185 (61.5%)
10. When did you use telerehabilitation for the first time?	After the pandemic	28 (24.1%)
	During the pandemic	57 (49.2%)
	Before the pandemic (COVID)	31 (26.7%)
11. Years of using telerehabilitation in your clinical practice.	< 5	96 (82.8%)
	5–10	17 (14.7%)
	11–15	2 (.1.7%)
	16–20	1 (0.9%)
12. How often do you use digital tools in clinical practice?	Everyday	27 (23.3%)
	Several Days per week	42 (36.2%)
	Several Days per month	47 (40.5%)
13. For what purpose do you use digital health tools/Telerehabilitation in clinical practice?	Patient appointment booking	57 (12.5%)
	Patient history taking	49 (10.8)
	Assessments (clinical tests/questionnaires)	52 (11.4%)
	Prescription of exercise program	69 (15.2%)
	Treatment delivery	34 (7.5%)
	Improve the treatment adherence (sending reminders)	41 (9%)
	Advice and information	80 (17.6%)
	Follow–up of treatment	73 (16%)
14. The most common app–based outcome measures.	Range of motion	53 (14.8%)
	Pain	58 (16.2%)
	Gait	40 (11.2%)
	Wound healing	7 (2%)
	Posture	52 (14.6%)
	Balance	41 (11.5%)
	Dexterity	51 (14.3%)
	Muscle strength	14 (3.9%)
	Respiratory	11 (3.1%)
	Other	30 (8.4%)

### Awareness and Perception

On a positive note, 85.3% of respondents revealed that incorporation of telerehabilitation during patient's management enhanced the quality of the care. More than 50% of the participants reported that telerehabilitation is a valid and reliable way to obtain outcome measures while almost 13% believed that telerehabilitation did not reliably or validly do so. In addition, 45.5% of the respondents agreed that telerehabilitation is not suitable for some pathological conditions.

As for the advantages of using digital tools, easy access to a physiotherapist, (particularly for patients who live in rural and remote areas) was noted by 67.2%, followed by time flexibility (60.5%) and cost savings (57.5%). In asking about the most common barriers of using telerehabilitation in Egypt, the most common answers were lack of awareness about digital health tools in society (59.9%), Internet connectivity issues (58.2%), and low technology literacy (55.9%).

A total of 82.9% of all participants reported that the effectiveness of telerehabilitation depends on the patient's expectation. Additionally, 81.9% recommended telerehabilitation to physiotherapists who don't use it (See Table 3).

**Table 3:** Awareness and Perception of Respondents to the Use of Telerehabilitation

Variable	Categories	Frequency
17. Do you believe that the inclusion of telerehabilitation would improve the quality of patient care?	Yes	255 (85.3%)
	No	44 (14.7%)
18. Do you believe that telerehabilitation provides reliable outcome measures?	Strongly not significant	1 (0.3%)
	Not significant	39 (13%)
	Neutral	98 (32.8%)
	Significant	123 (41.1%)
	Strongly significant	38 (12.7%)
19. Do you believe that telerehabilitation provides valid outcome measures?	Strongly not significant	1 (0.3%)
	Not significant	36 (12%)
	Neutral	100 (33.4%)
	Significant	123 (41.1%)
	Strongly significant	39 (13%)
20. Do you agree that telerehabilitation is NOT feasible or effective for certain patients?	Strongly agree	48 (16.1%)
	Agree	136 (45.5%)
	Neutral	68 (22.7%)
	Disagree	41 (13.7%)
	Strongly disagree	6 (2%)
21. What are the strengths or advantages of using digital health tools/telerehabilitation? (Multiple answers allowed.)	Cost savings	172 (57.5%)
	Easy access to a physiotherapist, particularly for patients who live in rural and remote areas	201 (67.2%)
	Flexible timetable	181 (60.5%)
	Treatment adherence	64 (21.4%)
	Outcome expectations	51 (17.1%)
	Better continuity of care for patients traveling (traveling patients)	159 (53.2%)
	Other	23 (7.7%)
22. What could be a barrier for using digital health tools/telerehabilitation in Egypt? (Multiple answers allowed.)	Provider willingness.	47 (15.7%)
	Low technology literacy	167 (55.9%)
	Internet connectivity issues	174 (58.2%)
	Lack of awareness about digital health tools/Telerehabilitation in society	179 (59.9%)
	Lack of connection between ICT (Information and Communication Technology) experts and clinicians	106 (35.5%)
	Inadequate staff skills	89 (29.8%)
	High costs.	69 (23.1%)
	Patients have difficulty in explaining their conditions to the therapist.	73 (24.4%)
	Patient privacy and difficulty in developing a relationship with the therapist	59 (19.7%)
	Patients’ adherence	49 (16.4%)
	Elderly or poorly educated patients	161 (53.8%)
	Others	23 (7.7%)
23. Do you agree that the expectations of the patients play a key role in shaping the effect of telerehabilitation results?	Agree	177 (59.2%)
	Strongly agree	71 (23.7%)
	Neutral	44 (14.7%)
	Disagree	7 (2.3%)
	Strongly disagree	(0.1%)
24. Do you think that telerehabilitation enhances your communication with your patients and allows you to have a more transparent interaction with them?	No	65 (21.7%)
	Yes	234 (78.3%)
25. Do you agree that digital health tools/telerehabilitation will play an essential role in the future of the profession?	Strongly agree	78 (26.1%)
	Agree	158 (52.8%)
	Neutral	52 (17.4%)
	Disagree	9 (3%)
	Strongly disagree	2 (0.7%)
26. Do you recommend digital health tools/telerehabilitation to other physiotherapists?	No	54 (18.1%)
	Yes	245 (81.9%)

### Attitude

A total of 172 (57.5%) of the participants reported that they are comfortable using telerehabilitation, and 62.6% stated that they could be more productive during telerehabilitation use. Also, 68.9% informed that data gathered during telerehabilitation is stored safely. Concerning incorporation of telerehabilitation in the students' training, 83.6% of the participants support the suggestion of giving telerehabilitation training during undergraduate education. Among the participants, 43.5% of PTs disagree that TR is wasting their time; 79% agreed that telerehabilitation systems could never replace in person consultation; and 52.6% reported that they perceive support from their colleagues to try out new digital tools (See Table 4).

**Table 4:** Attitude of Respondents to the Use of Telerehabilitation

Prompt: How much do you agree with the following statements?		
Variable	Categories	Frequency
27. I am comfortable with telerehabilitation applications.	Strongly disagree	3 (1%)
	Disagree	20 (6.7%)
	Neutral	104 (34.8%)
	Agree	136 (45.5%)
	Strongly agree	36 (12%)
28. I believe I could be more productive using telerehabilitation.	Strongly disagree	2 (0.7%)
	Disagree	19 (6.4%)
	Neutral	91 (30.4%)
	Agree	141 (47.2%)
	Strongly agree	46 (15.4%)
29. I believe that patient data gathered using digital tools is stored safely.	Strongly disagree	3 (1%)
	Disagree	18 (6%)
	Neutral	72 (24.1%)
	Agree	161 (53.8%)
	Strongly agree	45 (15.1%)
30. I think that physiotherapy students should be given formal training for telerehabilitation during their college education	Strongly disagree	1 (0.3)
	Disagree	7 (2.3%)
	Neutral	41 (13.7%)
	Agree	157 (52.5%)
	Strongly agree	93 (31.1%)
31. Due to the large number of patients in my practice, telerehabilitation is a waste of my valuable time.	Strongly disagree	14 (4.7%)
	Disagree	116 (38.8%)
	Neutral	103 (34.4%)
	Agree	53 (17.7%)
	Strongly agree	13 (4.3%)
32. Telerehabilitation can never replace in–person consultation.	Strongly disagree	2 (0.7)
	Disagree	8 (2.7%)
	Neutral	52 (17.4%)
	Agree	123 (41.1%)
	Strongly agree	114 (38.1%)
33. I perceive support from my colleagues regarding new digital tools for telerehabilitation.	Strongly disagree	4 (1.3%)
	Disagree	21 (7%)
	Neutral	117 (39.1%)
	Agree	135 (45.2%)
	Strongly agree	22 (7.4%)

## Discussion

The objectives of this study were to evaluate the utilization, awareness, perception, and attitude towards telerehabilitation among Egyptian physiotherapists. Most respondents in the present study were young adults and about two-thirds (65.9%) had experience of less than ten years. This finding is consistent with those observed in earlier studies that state that younger populations are more inclined towards smartphones, mobile apps, and social media platforms (Awotidebe et al., 2023; Dingli & Seychell, 2015; Mbada et al., 2021). This can be attributed to the various opportunities provided by the internet to younger generations including easy access to information, relationship building and maintenance, and rapid communication with others (Alvarez-Jimenez et al., 2014). Furthermore, younger people are more likely to adopt digital technologies than older people (Berkowsky et al., 2018). It is noteworthy that the majority of the participants included in the present study were working in teaching hospitals (42.5%) while the rest offered their services in settings like general hospitals (37.1%), private hospitals (35.5%), and other settings.

In the current study, less than 40% of physiotherapists reported using digital health tools at work and almost half first implemented telerehabilitation during the COVID-19 pandemic. These results seem to be consistent with those of Tsekoura et al. (2022) who found that most of the Greek physiotherapists engaged in telerehabilitation during the COVID–19 pandemic. On the other hand, Bezuidenhout et al. (2022) reported that telerehabilitation services were not delivered by most physiotherapists in Sweden before or during the pandemic.

Telerehabilitation includes services such as assessment, exercise program prescription, treatment delivery, follow up of treatment, advice, patient appointment booking and history taking. Many respondents use it almost equally for giving advice to the patients and for follow-up. A minority of respondents stated that they use telerehabilitation in treatment delivery. This finding is similar to a survey conducted by Rausch et al. (2021) in Switzerland who indicated that most physiotherapists use telerehabilitation in patient education and treatment follow-up.

Consistent with our study are the results of the systematic review by Mani et al. (2017) which concluded that there is good concurrent validity of the tele-applications used in the evaluation of pain, range of motion, balance, muscle strength, gait, posture, and dexterity. These were the most prevalent outcomes measured by the respondents. Only 3.9% used telepractice to measure muscle strength. Moreover, respiratory assessment as well as evaluating wound healing were the least common measurements.

Most respondent physiotherapists believed that telerehabilitation use would improve the quality of patient care. Furthermore, more than half of the respondents believed that telerehabilitation provides reliable and valid outcome measures. This finding is consistent with those of Aloyuni et al. (2020) who examined the implementation of telerehabilitation at various clinical settings across the kingdom of Saudi Arabia. In contrast, only about four of ten in Sweden realized that telerehabilitation would improve the quality of rehabilitation (Bezuidenhout et al., 2022). Moreover, a small percentage of UK physiotherapists expressed concerns about the accuracy and reliability of patient-reported outcomes assessed remotely (Buckingham et al., 2022). It is noteworthy that a recent systematic review concluded that digital physiotherapy assessments have acceptable to excellent validity and reliability (Bernhardsson et al., 2023).

Many Egyptian physiotherapists stated that the main barriers to telerehabilitation utilization are lack of awareness about digital health tools telerehabilitation (59.9%), low technology literacy (55.9%), Internet connectivity Issues (58.2%), patient privacy (19.7%) and elderly or poorly educated patients (53.8%). In a study conducted in Kuwait, 38% of the respondents reported that patient privacy could be a barrier (Albahrouh & Buabbas, 2021).

In Greece, most physiotherapists who participated in a study did not take any action regarding data privacy (Tsekoura et al., 2022) and security (Tsekoura et al., 2022). Jiménez-Rodríguez et al. (2020) also considered data privacy as a barrier due to technical difficulties and digital literacy by healthcare professionals and patients, especially elderly patients. Aloyuni et al. (2020) reported that technical issues are a barrier for 24% of the participants. According to Fernández-Lago et al. (2023) digital literacy is a challenge that should be solved to increase telepractice utilization.

Only 20% of the Saudi Arabian physiotherapists who participated in the Aloyuni, et al. (2020) study considered provider willingness as a barrier; 15.7% of the Egyptian PTs in the current study reported the same.

Albahrouh and Buabbas (2021) reported that the lack of connection between Information and Communication Technology (ICT) experts and clinicians was the most common barrier to telerehabilitation use. Only 35.5% of respondents in the current study reported it as a barrier.

Considering other barriers, our respondents reported that inadequate staff skills, high costs, and patients’ adherence are also challenging. In addition to patient privacy, patients evidenced difficulty explaining their conditions to the therapist and building a relationship with the therapist.

Aloyuni et al. (2020) mentioned the high cost of telerehabilitation utilization as a challenge; 23.1% of the participants in the present study agreed. Yet, 57.5% of our respondents believed telepractice is cost saving as did 83% of the participants in the Albahrouh and Buabbas (2021) study that noted that telepractice also saves time.

Easy access to a physiotherapist, particularly for patients who live in rural and remote areas was the most mentioned strength point of implementing tele-services besides better continuity of care for traveling patients. Treatment adherence was also believed to be positively affected by telerehabilitation utilization by 21.4% of the respondents.

Another interesting finding is that 61.1% of respondents perceived telerehabilitation as not feasible or effective for certain pathologies. This may be because the physiotherapy profession is perceived to deliver hands-on therapy (Malliaras et al., 2021; Rutberg et al., 2013). This finding agrees with the opinion of UK rehabilitation practitioners who acknowledged that telerehabilitation may not be suitable for every case and that remote consultation is less appropriate with elderly persons, individuals with severe sensory, cognitive, or physical impairments, and when manual prosthetic adjustment is required (Buckingham et al., 2022). Most of the participants agreed that patients’ expectations play a key role in shaping the effect of telerehabilitation results. Lower patients’ expectations were proposed as a potential challenge to the adoption and usage of telerehabilitation (Peterson et al., 2022).

Considering patient-therapist communication, most therapists (78.3%) indicated that telerehabilitation allows for more enhanced transparency and improves their communication with patients. Consistent with our finding, a group of tele-experienced health professionals emphasized that their communication skills improved and that they had eventually been able to connect and engage with the patients online (Damhus et al., 2018). Further, participants who were enrolled in a telerehabilitation program were satisfied with video calls/messaging apps communication (Bernal-Utrera et al., 2021). This is in contrary to findings of a focused group discussion study conducted in Malaysia in which participant physiotherapists, irrespective of their experience level, raised concerns about communication obstacles that may emerge during the implementation of telerehabilitation. Their main concerns included misunderstanding of instructions, less patients’ active engagement, and difficulty in remembering appointments when using digital health tools (Sia et al., 2024).

In the current study, most respondents agreed that digital health tools/telerehabilitation will play an essential role in the future of the profession. Accordingly, they recommended digital health tools/telerehabilitation to other physiotherapists. This may increase digital awareness among other colleagues who might not have been as willing to try out novel forms of intervention (D’Souza & Rebello, 2021).

Our results showed that 57.5% of respondents felt comfortable using telerehabilitation applications, a finding in agreement with a prior study conducted by Albahrouh and Buabbas (2021). In the latter study, most Saudi physiotherapists felt comfortable using telerehabilitation and therefore intended to use telehealth. They perceived that telehealth benefited their working routine and resulted in positive modifications. In contrast, a previous study conducted by Rowe and Saul (2020) reported that the majority of South Africa physiotherapists did not feel comfortable prescribing apps to patients for their own use.

Our survey data showed that 43.5% of physiotherapists disagreed that telerehabilitation wastes their time. This is consistent with the survey findings of D'Souza and Rebello (2021) in India. They reported that 62.6% of their sample believed that telerehabilitation technology makes them more productive and enables saved time. This outcome is consistent with a study of physiotherapists conducted by Awotidebe et al. (2023) in Nigeria in which a majority believed they could be more productive using telerehabilitation.

Important issues in the conduct of telerehabilitation are data security and patient confidentiality. Our survey participants (69%) agreed that patient data gathered using digital tools was stored safely. This finding is in contrast with a study conducted by Albahrouh et al. (2021) in Kuwait in which patient privacy and the confidentiality of their data were significant concerns that the physiotherapists considered as barriers to the use of telerehabilitation.

Most respondents (84%) expressed that physiotherapy students should be given formal training in telerehabilitation during their university education. This finding is in line with the work of Michell et al. (2022), who reported that the University of Queensland established the first telerehabilitation interdisciplinary student clinic in Australia in 2015 after realizing that students needed practical clinical experience and education in the use of technology for clinical service delivery. Also, D'Souza and Rebello (2021) emphasized the importance of providing training, as a lack of training in telerehabilitation practice was one of the most commonly reported barriers.

The findings show consensus agreement among the respondents (79%) that telerehabilitation could never totally replace in-person consultation. This finding is in line with Albahrouh and Buabbas's (2021) study in Kuwait. Physiotherapy is a hands-on, physically present profession, and there was concern that it can be challenging to use telerehabilitation devices to undertake some examinations and treatments. Also, a small percentage of Greek PTs prefer to practice physiotherapy via conventional in-person methods (Tsekoura et al., 2022).

In the current study, telerehabilitation was deemed appropriate in the workplaces of most participants. Additionally, 52.6% of respondents perceived support from their colleagues to try out new digital tools.

## Limitations

To our knowledge, this is the first study to investigate physiotherapists’ utilization, awareness, perceptions and attitudes toward telerehabilitation in Egypt. The following limitations should be considered. First, the study's findings rely on self-reported responses; thus, over generalization of the physiotherapists’ responses might have occurred. Second, the study does not include patients’ knowledge and attitudes toward telerehabilitation.

## Future Directions

The future use of telerehabilitation by physiotherapists in Egypt is promising, with the potential to expand its reach and impact. Incorporating technology and telerehabilitation courses into undergraduate and postgraduate curricula will provide future physiotherapists with the necessary skills to successfully utilize digital health tools. Raising patients’ and physiotherapists’ awareness about the benefits of telerehabilitation as a feasible alternative or complement to in-person therapy will promote its adoption. Finally, investing in improved internet infrastructure and training in digital health technologies for practicing physiotherapy professionals will facilitate the widespread use of telerehabilitation services.

## Conclusion

In general, Egyptian physiotherapists expressed an optimistic attitude toward the use of telerehabilitation. Yet, few had implemented telerehabilitation services before or during COVID-19. They also identified multiple barriers to its implementation. As a result, the findings of this study highlight the trends and challenges in utilizing telerehabilitation and are poised to contribute to shaping its future in Egypt.

## Appendix

### Survey

**Dear Colleague,**

It is our pleasure to invite you to take part in this survey, which is a research study aiming to investigate “the application, and opinion of physiotherapists towards tele-rehabilitations in Egypt”.

We hope to answer all the questions accurately and objectively. It may take 10–15 minutes to complete the questionnaire.

Your cooperation is highly appreciated.

**Digital health** is commonly used as an umbrella term describing the use of information and communication technology in support of health and health-related fields, including smartphone applications, wearable sensors, activity tracking devices, and telehealth platforms.

**Tele-rehabilitation** has been defined as the delivery of rehabilitation services via information and communication technologies to people remotely in their environment. These services include assessment, prevention, treatment, education, and counselling.

**Please click the following indicating your choice to be in this study:**

○ Yes, I agree to participate in the study.
○ No I do not want to participate in the study

**Sociodemographic Data**

**1- Gender:**
○ Male
○ Female
**2- Age:**
○ 23–30 years
○ 31–39 years
○ 40–49 years
○ 50–59 years
○ ≥60 years
**3- Governate:**
○ Alexandria
○ Great Cairo (Cairo - Giza - Qalubia)
○ Delta Governorates
○ Upper Egypt Governorates
○ Canal City Governorates
○ Sinai and Red Sea
○ Other
**4- Highest Physical Therapy Degree (Level of Education):**
○ Bachelor’s
○ Diploma
○ Master’s
○ Doctor of Philosophy (PhD)
○ Doctor of Physical Therapy (DPT)
**5- Work setting (Multiple answers allowed):**
○ Teaching hospitals/institute
○ General hospital
○ Private
○ Other
**6- Area of expertise (Multiple answers allowed):**
○ Cardiothoracic
○ Ergonomics
○ Geriatric rehabilitation
○ Integumentary and post-burn rehabilitation
○ Neurology
○ Nutrition
○ Oncology
○ Orthopaedics
○ Paediatrics
○ Sports
○ Women's Health
○ Not applicable
**7- Working experience:**
○ < 5 years
○ 5–10 years
○ 11–15 years
○ >15 years
**8- How many actual hours per week do you work?**
○ <10
○ 10–20
○ 21–30
○ 31–40
○ >40

**Utilization of tele-rehabilitation Services**

**9- Do you use digital health tools/tele-rehabilitation at work?**
○ Yes
○ No

**If your answer is YES, for the previous question, then answer questions 10–15. But if no, answer question 16.**

**10- When did you use tele-rehabilitation for the first time?**
○ Before the pandemic (COVID)
○ During the pandemic
○ After the pandemic
**11- Years of using tele-rehabilitation in your clinical practice:**
○ < 5
○ 5–10
○ 11–15
○ 16–20
**12- Which digital tools do you use in clinical practice? (Multiple answers allowed)**
○ Telephone/SMS services
○ video conferencing
○ internet-based application/social media
○ mobile applications
○ virtual reality
○ Others
**13- How often do you use digital tools in clinical practice?**
○ Every day
○ Several days per week
○ Several days per month
**14- For what purpose do you use digital health tools/tele-rehabilitation in clinical practice? (Multiple answers allowed).**
○ Patient appointment booking
○ Patient history taking
○ Assessments (clinical tests/questionnaires)
○ Prescription of exercise program
○ Treatment delivery
○ Improve the treatment adherence (sending reminders)
○ Advice and information
○ Follow-up of treatment
**15- The most common app-based outcome measures (Multiple answers allowed):**
○ Range of motion
○ Pain
○ Gait
○ Wound healing
○ Posture
○ Balance
○ Dexterity
○ Muscle strength
○ Respiratory
○ Other
**16- If your answer is no, to question 9, why did you not offer tele/online therapy?**
○ I was able to provide my patients with sufficient care in another way.
○ I cannot observe the patient adequately.
○ I miss the hands-on experience.
○ The technical possibilities are unknown to me or my patients.
○ Other reasons

**Awareness and Perception**

**17- Do you believe that the inclusion of tele-rehabilitation would improve the quality of patient care?**
○ Yes
○ No
**18- Do you believe that tele-rehabilitation provides reliable outcome measures?**
○ Yes
○ No
**19- Do you believe that tele-rehabilitation provides valid outcome measures?**
○ Yes
○ No
**20- Do you agree that tele-rehabilitation is NOT feasible or effective for certain patients?**
○ strongly agree.
○ Agree
○ Neutral
○ Disagree
○ Strongly disagree.
**21- What are the strengths or advantages of using digital health tools/Tele-rehabilitation? (Multiple answers allowed)**
○ Cost savings
○ Easy access to a physiotherapist, particularly for patients who live in rural and remote areas.
○ Flexible timetable
○ Treatment adherence
○ Outcome expectations
○ Better continuity of care for patients traveling (traveling patients)
○ Others
**22- What could be a barrier for using digital health tools/tele-rehabilitation in Egypt? (Multiple answers allowed)**
○ Provider willingness.
○ Low technology literacy
○ Internet connectivity issues
○ Lack of awareness about digital health tools/tele-rehabilitation in society
○ Lack of connection between ICT (Information and Communication Technology) experts and clinicians
○ Inadequate Staff skills
○ High costs.
○ Patients have difficulty in explaining their conditions to the therapist.
○ Patient privacy and difficulty in developing a relationship with the therapist
○ Patients’ adherence
○ Elderly or poorly educated patients
○ Others
**23- Do you agree that the expectations of the patients play a key role in shaping the effect of tele-rehabilitation results?**
○ strongly agree.
○ Agree
○ Neutral
○ Disagree
○ Strongly disagree.
**24- Do you think that tele-rehabilitation enhances your communication with your patients and allows you to have a more transparent interaction with them?**
○ Yes
○ No
**25- 24-Do you agree that digital health tools/tele-rehabilitation will play an essential role in the future of the profession?**
○ Strongly agree.
○ Agree
○ Neutral
○ Disagree
○ Strongly disagree.
**26- Do you recommend digital health tools/tele-rehabilitation to other physiotherapists?**
○ Yes
○ No

**Attitude**

**How much do you agree with the following statements?**

**27- I am comfortable with tele-rehabilitation applications.**
○ Strongly agree.
○ Agree.
○ Neutral
○ Disagree.
○ Strongly disagree.
**28- I believe I could be more productive using tele-rehabilitation.**
○ Strongly agree.
○ Agree.
○ Neutral
○ Disagree.
○ Strongly disagree.
**29- I believe that patient data gathered using digital tools is stored safely.**
○ Strongly agree.
○ Agree.
○ Neutral
○ Disagree.
○ Strongly disagree.
**30- I think that physical therapy students should be given formal training for tele-rehabilitation during their college education.**
○ Strongly agree.
○ Agree.
○ Neutral
○ Disagree.
○ Strongly disagree.
**31- Due to the large number of patients in my practice, tele-rehabilitation is a waste of my valuable time.**
○ Strongly agree.
○ Agree.
○ Neutral
○ Disagree.
○ Strongly disagree.
**32- Tele-rehabilitation can never replace face-to-face consultation.**
○ Strongly agree.
○ Agree.
○ Neutral
○ Disagree.
○ Strongly disagree.
**33- I perceive support from my colleagues regarding new digital tools for tele-rehabilitation.**
○ Strongly agree.
○ Agree.
○ Neutral
○ Disagree.
○ Strongly disagree.
**End of questionnaire, Thank you**
